# Ocular injuries associated with motor vehicle accidents: long term effects on quality of life

**DOI:** 10.1007/s10792-024-03083-z

**Published:** 2024-03-14

**Authors:** Judith Brody, Meydan Ben Ishai, Irena Serov-Volach, Keren Mano-Tamir, Dan D. Gaton, Inbal Avisar

**Affiliations:** 1 Department of Ophthalmology, Rabin Medical Center, 39 Jabotinski St., 49100 Petah Tikva, Israel; 2 The Faculty of Medicine, Tel Aviv University, Tel Aviv, Israel

**Keywords:** Eye injury, Vision, Motor vehicle accidents, Recovery, Daily activities

## Abstract

**Purpose:**

To describe the prevalence and outcome of motor vehicle accidents-associated ocular injuries.

**Methods:**

A survey of patients who presented to the emergency room at a level 1 trauma center with motor vehicle accidents-associated ocular injuries. A patient questionnaire and review of clinical notes were conducted for all patients.

**Results:**

Of 274 motor vehicle accident victims with ocular injuries who presented to the emergency room, 40 (15%) responded to the survey. Over half of them were driving a vehicle, and most reported wearing a seat belt or a helmet. Most ocular injuries were mild. The most common injuries were bone fractures, subconjunctival hemorrhage, eyelid involvement and corneal injury. Most respondents had no change in vision and perceived their ocular involvement as a minor part of their injury. Most respondents returned to work and to driving within a year.

**Conclusion:**

Our study sheds light on the details and extent of ocular involvement and the visual ability to perform daily activities following motor vehicle accidents.

## Introduction

Traumatic injuries due to motor vehicle accidents (MVA) are a common global occurrence and the leading cause of death among children and young adults aged 5–29. About 1.35 million people die each year due to MVAs [1]. Head and facial injuries are commonly associated with high rates of ocular injury and resultant morbidity [2]. MVAs present specific risk factors for eye injuries, including exposure to broken glass and other foreign bodies, rapid changes in velocity, lack of passenger restraint such as seatbelts, and airbag deployment [3–7]. Ocular injury is the second leading cause of monocular blindness in the United States, second only to cataracts as the most common cause of visual impairment [8]. An evaluation of the characteristics of open globe injuries following road accidents showed that most patients suffered from rupture, while only 20% had penetrating eye injuries [9]; only 40% of patients reported using a seat belt in that study [9]. A review of the impact of vehicle occupant restraint systems on eye injuries showed that airbags could lead to corneal abrasions, alkali burns, and globe compression [10]. The occupational, financial, and psychosocial consequences of road injuries carry a personal and public burden, which reduces the injured individual's quality of life [11]. Assessment of long-term visual and social outcomes of patients with ocular injuries is essential for describing the burden of MVA; however, the literature lacks studies that directly address these issues [10]. In this study, we aimed to describe the prevalence and outcome of MVA-associated ocular injuries in a tertiary medical center in the country. We emphasize the impact of ocular injuries regarding visual tasks and functional outcomes on long-term follow-up.

## Materials and methods

### Study design and population

We analyzed medical charts of patients who presented to the emergency room at a level 1 trauma center in the country with MVA-associated ocular injuries between 2000 and 2010 and were referred to the ophthalmology department for evaluation.

The Institutional Review Board of Rabin Medical Center approved the study and conducted it according to its rules and regulations, following the tenets of the Declaration of Helsinki.

A telephone survey was conducted at a minimum of 3 years following the accident to assess the characteristics of each accident. The respondents were asked about the MVA: the type of vehicle, if the patient was a driver, passenger (behind or next to the driver), or pedestrian, and the safety measures at the time of the accident (helmet, seatbelt, airbag deployment). The respondents were also interrogated about visual difficulties performing daily activities such as reading, watching television, and operating a computer. The time lap from the accident until returning to work and driving following the injury was also documented. The Ethics Committee of this medical centre has approved the study. Written informed consent from patients was not required.

### Statistical analysis

The data were analyzed using the SAS® version 9.3 (SAS Institute, Cary, North Carolina). Statistical analysis included descriptive statistics with continuous variables summarized using the arithmetic mean, standard deviation, median, minimum and maximum values, and absolute values summarized using absolute and relative frequencies. Cross-tables were generated by safety measures, accident type, and fracture outcome, and the chi-squared test was applied to analyze the difference in proportions for the above parameters. *P* values of less than 0.05 were considered statistically significant.

## Results

Over ten years, 274 MVA victims with ocular injuries presented to the ER. Of these, 40 individuals (15%) responded to the telephone survey. The reasons for non-response, respondent demographics and MVA characteristics are presented in Table 1.

**Table 1:** Participant demographics and MVA characteristics

	Survey respondents N = 40
Median age, years (range)	26.5 (17.0–67.0)
*Gender, n (%)*	
Males	25 (62.5%)
Females	15 (37.5%)
*Vehicle type*	
Private	38 (95%)
Commercial	2 (5.0%)
*Respondent’s role during the MVA*	
Car driver	14 (32.5%)
Motorcycle driver	6 (15%)
Bicycle rider	1 (2.5%)
Car passenger	13 (32.5%)
Pedestrian	5 (12.5%)
*Safety measures during the accident*	
Seatbelt	17 (42.5%)
Airbag deployment	7 (17.5%)
Helmet	5 (12.5%)
None	11 (27.5%)

The mean time interval between the occurrence of injury and questionnaire response was 9 years. Of the 40 responders to the telephone survey, 27 responders (67.5%) were in a car when the accident occurred; 17 (42.5%) reported wearing seat belts, and seven (17.5%) reported that the car's airbags had been deployed. Of the seven respondents (17.5%) who reported riding a motorcycle or a bicycle, five (12.5%) wore a helmet at the time of the accident. The characteristics of ocular injuries are shown in Table 2.

**Table 2:** Ocular Injury Characteristics

	Respondents N = 40^^^
Fractures^§^	27 (67.5%)
Lateral orbital wall fracture	16 (59.3%)*
Orbital roof fracture	12 (44.4%)*
Orbital floor fracture	11 (40.7%)*
Medial orbital wall fracture	6 (22.2%)*
Optic canal fracture	2 (7.4%)*
Subconjunctival hemorrhage	16 (40.0%)
Eyelid cut	9 (22.5%)
Cornea injury	5 (12.5%)
Retinal injury	3 (7.5%)
Ptosis	2 (5.0%)
Perforation	1 (2.5%)

^**^**^19 patients (47%) had more than one ocular injury

*Percentage out of 27 patients who suffered fractures

^**§**^13 patients, representing 48% out of 27 patient who suffered fractures, had more than one type of fracture

Most respondents returned to work within a year after the accident (Table 3).

**Table 3:** Time to return to work/studies and to driving

	Respondents N = 40
Returned to work/studies	25 (62.5%)
Within 1 month	2 (5.0%)
Within 3 months	1 (2.5%)
Within 6 months	1 (2.5%)
After more than 6 months	7 (17.5%)
Within 1 year	14 (35.0%)
Returned to driving	28 (70.0%)
Within 1 month	5 (12.5%)
Within 6 moths	4 (10.0%)
After more than 6 months	5 (12.5%)
Within 1 year	14 (35.0%)

Twenty-two patients (55%) sustained severe systemic injuries, including loss of consciousness, severe head injury, and hospitalization in intensive care and neurosurgery units. Three patients had both severe systemic injury and severe ocular injury.

Most respondents (28, 70%) also resumed driving within a year, nine of which (22.5%) returned to driving within 6 months following the accident. The time to return to work, academic activity or driving was not associated with the safety measures taken while driving (seatbelt, helmet or airbag) or the type of vehicle (car, motorcycle, bicycle or pedestrian) or the occurrence of fractures (data not shown). Most respondents (70%) had no change in vision following their injury and perceived their ocular involvement as a minor part of their MVA injury. Only a minority of patients had a visual decline, according to patient reports (Table 4).

**Table 4:** Ocular injury outcome as reported by patients

	Respondents N = 40
Blurry vision	9 (22.5%)
Headache	8 (20.0%)
Difficulties in watching television	5 (12.5%)
Convergence insufficiency	4 (10.0%)
Reading difficulties	2 (5.0%)
Epiphora	2 (5.0%)
Blindness	1 (2.5%)

Safety measures are taken while driving (seatbelt, helmet or airbag) were not associated with the occurrence of orbital fracture (*P* < 0.12), injury severity (*P* < 0.21) or change in vision (*P* < 0.29).

The type of vehicle (car, motorcycle, bicycle or pedestrian) was not associated with the occurrence of orbital fracture (*P* < 0.6), injury severity (*p* < 0.23), or change in vision (*p* < 0.33).

## Discussion

Our study showed that most patients presenting with eye injury following MVA were young (median age: 26.5 years) and males. These findings align with other reports showing that males are at a higher risk of being injured in MVA than females [5, 12–14]. Over half of the respondents in our cohort were driving the vehicle at the time of the accident. This finding aligns with other studies that reported a higher eye injury rate among vehicle drivers than passengers [3–5, 12]. Most respondents reported wearing a seat belt or a helmet during the accident. Mandatory seatbelt laws have lowered the incidence of penetrating eye injuries in road traffic accidents [15, 16]. Still, it was suggested that in the event of a frontal airbag deployment, front-seat occupants of motor vehicles that sustained a frontal collision were at a two-fold risk of experiencing an ocular injury, including intraocular hemorrhages, corneal injuries, hyphema, detachments and retinal tears [5, 17].

A systematic review of the effectiveness of restraining devices on eye injury in motor vehicle collisions showed that using seat belts decreased eye injuries in motor vehicle collisions. At the same time, airbags had no significant effect on increased rates of eye injuries [18]. These findings may be due to newer airbag technology with reduced inflation force [19]. However, it was suggested that significant sight-threatening ocular injuries related to airbags, such as traumatic airbag maculopathy [20], can occur despite the lack of apparent external trauma to the eye [19]. Our analysis did not show an association between the use of seat belts or the deployment of airbags and the severity of an ocular injury or change in vision following the injury. The discrepancy between the results of our study and previous reports regarding the use of seatbelts or airbags and the seriousness of eye injuries may be related to selection bias; it is plausible that the most severe cases of eye injuries did not answer the questionnaire due to other significant morbidities. Patients with other systemic injuries may not percept their ocular injury as significant due to other health issues. Most of the ocular injuries (90%) described in our study were mild, consistent with other reports on ocular injuries following MVA [5, 13]. The most common type of ocular injury was orbital bone fracture (67.5%). The incidence of MVA-associated facial fractures, including nasal and orbital fractures, in the US, was 10.9% [21]. The higher rates noted in our cohort may be partly explained by the 15% response rate to the survey and to the fact that our study was conducted only in a level 1 trauma referral center, in contrast to various US trauma centers, including levels 1, 2, 3 and 4, in the study mentioned above. Our results showed that among orbital fractures, the lateral wall was the most common site of insult (59.3%), followed by a roof (44.4%), floor (40.7%), medial wall (22.2%) and optic canal (7.4%) involvement. Another study reported that the most frequent isolated orbital fracture because of MVA was a floor fracture, followed by medial, roof, and lateral wall fractures [22]. A retrospective cross-sectional study in the US, using the National Electronic Injury Surveillance System All Injury Program from 2001 to 2008 to assess the risk of presenting to an ED with an MVA- associated eye injury reported that the most common diagnoses were contusion/abrasion (61.5%), foreign body (19.7%) and hemorrhage (4.1%) [12]. The most common ocular injuries presenting to a northern regional medical center between 2007 and 2011 were subconjunctival hemorrhage (34%), corneal erosions (27%), hyphemia (20.3%) and lid lacerations (18%), while orbital fractures constituted only 0.9% [13].

According to a report by the US National Highway Traffic Safety Administration, the economic cost of MVA-associated injuries is enormous, with cost components including productivity losses, property damage, medical expenses, rehabilitation costs, congestion costs, legal and court costs, emergency services such as medical, police, and fire services, insurance administration costs, and the costs to employers [23]. Road traffic crashes cost most countries three percent of their gross domestic product [1]. MVA also carry a psychosocial burden on the patients and reduces their quality of life compared to the general population norms [11]. Our results showed that although the ocular injuries were considered mild and only 30% of patients regarded the MVA-associated ocular injury as a major factor following the accident, the injuries substantially affected the respondents' ability to return to work. Only four respondents (10%) returned to work within six months following the accident, whereas 62.5% returned within one year. The remaining respondents reported a continued absence from work. Even minor injuries following MVA were reported to have a major impact on returning to work and pre-injury participation during the two years after the injury, as well as on the health-related quality of life and physical and mental well-being [24, 25]. Concerning vision, only a minority of our patients had visual decline resulting in reading difficulties (5%), watching television difficulties (12.5%) and blindness (2.5%).

This study's limitations include its retrospective nature; however, we added a prospective aspect by actively contacting patients to determine the long-term consequences of their traumatic injury. This information is valuable in reflecting the impact of quality of life and social burden. The average follow-up spans over nine years and therefore entails a more precise reflection of long-term sequelae. Although a relatively large prospective quality-of-life study may be required for statistical proof, it offers information that can motivate elaborating preventive measures against ocular motor vehicle injuries to reduce their incidence. One of the challenges in the current study was to isolate the ocular injuries from other body injuries of the MVA patients and to assess their functional impact. The data was collected in one of the largest medical centers in the country and thus represented an approximation of the actual incidence of these types of injuries across the country.

In conclusion, our study sheds light on the details and extent of ocular involvement and the visual ability to perform daily life activities following MVA. Although ocular injuries were considered mild and less than a third of the patients regarded the MVA- associated ocular injury as a major factor following the accident, the injuries substantially affected the respondents' ability to return to work or driving. Further research is needed to evaluate our findings in a larger population by increasing the response rate to the survey.
